# A general framework for selecting work participation outcomes in intervention studies among persons with health problems: a concept paper

**DOI:** 10.1186/s12889-022-14564-0

**Published:** 2022-11-26

**Authors:** Margarita Ravinskaya, Jos H. Verbeek, Miranda W. Langendam, Ira Madan, Suzanne M. M. Verstappen, Regina Kunz, Carel T. J. Hulshof, Jan L. Hoving

**Affiliations:** 1 Amsterdam UMC, Academic Medical Center, Department of Public and Occupational Health, Coronel Institute of Occupational Health, University of Amsterdam, Amsterdam Public Health research institute, Amsterdam, The Netherlands; 2 Amsterdam UMC, Academic Medical Center, Department Epidemiology and Data Science, University of Amsterdam, Amsterdam Public Health research institute, Amsterdam, The Netherlands; 3 Guy’s and St Thomas’ NHS Trust and Faculty of Life Sciences and Medicine, King’s College London, Centre for Musculoskeletal Health and Work, London, United Kingdom; 4 Centre for Epidemiology Versus Arthritis, Faculty of Biology, Medicine and Health, The University of Manchester, Manchester, United Kingdom; 5 NIHR Manchester Biomedical Research Centre, Manchester University NHS Foundation Trust, Manchester Academic Health Science Centre, Manchester, United Kingdom; 6 MRC Versus Arthritis Centre for Musculoskeletal Health and Work, University of Southampton, Southampton, United Kingdom; 7 Research Unit EbIM, Evidence Based Insurance Medicine, Division of Clinical Epidemiology, University Hospital and University of Basel, Basil, CH Switzerland

**Keywords:** Work participation, Return to work, Sick leave, Employment, Occupational functioning, Work ability, Outcome measurement, Occupational health, Vocational rehabilitation, Research framework, core outcome set

## Abstract

**Background:**

Work participation is important for health and can be considered as engagement in a major area of life which is of significance for most people, but it can also be thought of as fulfilling or discharging a role. Currently, academic research lacks a comprehensive classification of work participation outcomes. The International Classification of Functioning is the foremost model in defining work functioning and its counterpart work disability, but it does not provide a critical (core) set of outcomes. Standardizing the definitions and nomenclature used in the research of work participation would ensure that the outcomes of studies are comparable, and practitioners and guideline developers can better decide what works best. As work participation is a broad umbrella term including outcome categories which need unambiguous differentiation, a framework needs to be developed first.

**Aim:**

To propose a framework which can be used to develop a generic core outcome set for work participation.

**Methods:**

First, we performed a systematic literature search on the concept of (work) participation, views on how to measure it, and on existing classifications for outcome measurements. Next, we derived criteria for the framework and proposed a framework based on the criteria. Last, we applied the framework to six case studies as a proof of concept.

**Results:**

Our literature search provided 2106 hits and we selected 59 studies for full-text analysis. Based on the literature and the developed criteria we propose four overarching outcome categories: (1) initiating employment, (2) having employment, (3) increasing or maintaining productivity at work, and (4) return to employment. These categories appeared feasible in our proof-of-concept assessment with six different case studies.

**Conclusion:**

We propose to use the framework for work participation outcomes to develop a core outcome set for intervention studies to improve work participation.

**Supplementary Information:**

The online version contains supplementary material available at 10.1186/s12889-022-14564-0.

## Introduction

According to the World Disability Report of 2011, about 978 million adults experience a form of disability which impairs their functioning in daily life [1]. Having a disability may have a negative impact on well-being due to associated social isolation, poor mental health and strained family relationships [2]. Societal repercussions include government expenditure on disability benefits, reduced work capacity in terms of attendance and productivity and early retirement schemes which in total may account for ~ 2% of GDP in OECD countries [1]. Even though paid work participation is a concern on the socioeconomic level, it is particularly important in the fields of rehabilitation and occupational health. In research, much effort is directed at interventions to increase work participation in people with ill health [3].

Systematic reviews provide insight into which interventions are beneficial and help guide future guidelines, practice and policy [4]. However, researchers and authors of systematic reviews on work participation in relation to health have reported the challenges of heterogeneous outcome measurement in trials which impedes large-scale evidence comparison [5–9]. Variability in the use of terminology is one of the reasons why comparison is currently difficult [10, 11]. Work participation and work disability are not clearly defined terms and are used with varying meanings. To resolve communication issues, the WHO developed the International Classification of Functioning, Disability and Health (ICF) [12]. In the ICF, participation in general is defined as involvement in a life situation. Work and employment are major life situations in which people should be able to participate in spite of ill health [13]. Following Alheresh and Keysor’s interpretation of the ICF [14], work functioning is defined as an overarching term for work activities and work participation, and work disability as a limitation of work activities or a restriction of work participation. This makes work disability a broad term denoting that people with impaired health have difficulty in performing work related tasks or maintaining employment.

Although vocational rehabilitation and occupational health fields use different terms, the aims and general content of interventions are the same. However, clear distinctions need to be made for outcome categories. Studies evaluating the effects of drug therapy on work productivity require different work outcome measures than studies evaluating supported employment interventions enabling unemployed people to enter the job market [10]. We have previously shown extensive variability in the use of outcome measurements across studies evaluating work outcomes [10].

Core Outcome Sets (COS) are recommended as a solution to heterogeneous outcome measurements [15]. A COS is an agreed set of minimum outcomes which should be measured in all trials in a specific health area. Only the consistent use of COS will allow large-scale pooling of outcomes and decrease outcome reporting bias [16]. One of the most prominent examples of the development of a COS is the Outcome Measures in Rheumatology Initiative (OMERACT). Over the past 30 years, a group of researchers in the field of rheumatology has worked on recommending or developing a range of outcome measurements including work participation for people with rheumatic and musculoskeletal diseases [17]. Due to this initiative, unambiguous outcome reporting of randomized controlled trials (RCTs) within rheumatology has considerably improved [15, 18].

Many authors have discussed conceptual issues of measuring work participation through a range of perspectives, but no overview exists [19–23]. To date, no COS exists for work participation outcomes that can be used for intervention studies to improve work participation for people experiencing health problems. To that end, we started a project to develop a generic COS for work participation (COS for Work) (http://www.cosforwork.org/).

Our systematic review on how work outcomes are measured across disciplines found two main categories of interventions that use work participation for their evaluation [10]. Studies that report vocational interventions aim to directly improve work-related or work ability related factors. Medical or clinical studies that report non-vocational interventions aim to indirectly impact work participation by improving clinical features and use symptoms or signs as their primary outcome and measure work participation improvements as a secondary outcome. The scope of the COS for Work includes outcomes that measure the success of all kinds of interventions aimed at impacting work participation whether these are vocational or clinical or a combination of the two.

Before developing a COS, it is important to understand what factors to consider when selecting and measuring work participation outcomes. In this initial stage, we aim to propose a framework for the development of the generic COS.

The objectives of this concept paper are:

i) to provide an overview of theoretical perspectives on work participation interventions and their outcome measurements, ii) to evaluate the literature for classifications of work participation outcomes, iii) to derive criteria for classifying work participation outcomes, and iv) to propose a framework to support the selection of work participation outcomes for interventions aiming to help people with (general or specific) health problems and to test the framework as proof of concept.

## Methods

**Fig. 1:**
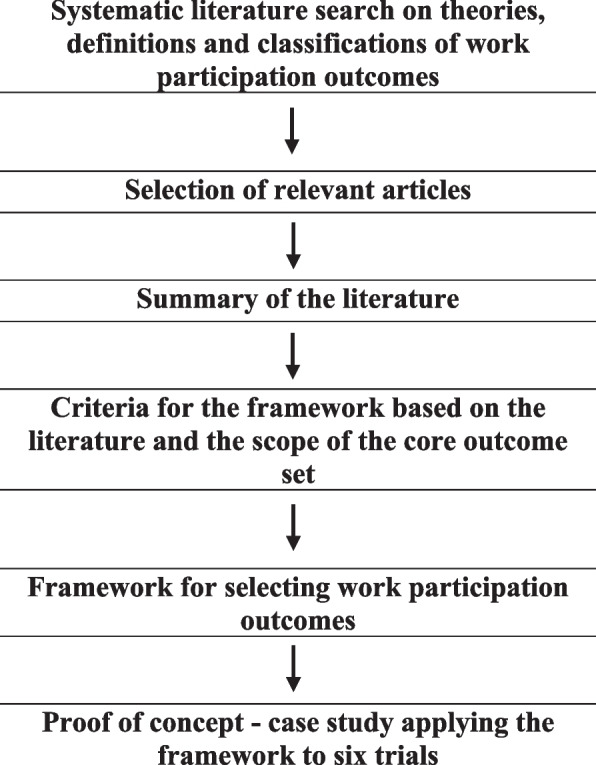
Steps taken to create the framework

We followed the guidance on writing conceptual papers [24, 25] which aim to bridge existing theories, provide multi-level insights, broaden the scope of our thinking or provide or adapt theories or result in a new typology. The concept of this paper focusses on a framework for typifying existing work participation outcomes. We used existing concepts and theories that we located through a systematic literature search as the input for our analysis. The findings were synthesized in a narrative way. To identify previous studies on definitions or classifications of work participation outcomes (Fig. 1), we conducted a systematic literature search and used the following eligibility criteria to select articles: the study should (1) include people who either work or want to work (more), (2) focus on change in work participation due to an intervention, and (3) refer to a theory, framework, model or categorization of work participation outcomes or elements of work participation. We constructed a search strategy in MEDLINE (PubMed) (Additional file 1: search strategy) and searched from inception (1966) up to 22/10/2021. Grey literature was searched using the general search engine Google. Because Google has its own search algorithm, we only used the search terms sick leave, sickness absence, work ability, or disability in combination with theory, concept or classification. As Google sorts according to relevance, we explored the first 20 hits further. In addition, we searched our personal files for relevant studies. One author (JV) removed references that did not fulfil the inclusion criteria. Three authors (MR, JV, JH) then selected references for full-text assessment and inclusion based on the full text. We extracted data on whether the article described a classification, the name of the classification, and the specific classification or outcome addressed.

Using the selected studies, we summarized existing theories that described (work) participation in general, classifications of work outcomes and current definitions of specific work outcomes. Next, to build our framework we identified criteria that were derived from the literature and described these. We used the criteria to create a new framework that can serve as a starting point for selecting work participation outcomes for the COS for Work. The elements for this framework were developed using COS methodology from COMET initiative [16] and was guided by the scope of the COS for Work (i.e., outcomes relevant for any type of intervention aiming to impact work participation for individuals with any type of health problem). Based on the identified literature one author (MR) drafted a proposal of the criteria which were then disused by all authors during three online meetings to improve the terminology, to evaluate priorities and to discuss to what degree these criteria were feasible to implement. All authors participated in group discussions and provided written comments. The authors cover a wide area of expertise such as occupational medicine, physiotherapy, occupational therapy, epidemiology and systematic review methodology.

Finally, as a proof of concept, we performed a case study where we applied the framework of work participation outcomes to six RCTs that constituted a representative sample of short-term, intermittent and long-term health conditions studied in work participation research. This step was important as COS for Work is not disease specific and should be relevant for all types of health problems.

We took the following approach to assess / determine proof of concept: First, we identified six common health conditions with different courses of disease typically impacting work participation (schizophrenia, depression, rheumatoid arthritis, breast cancer, influenza, various health problems causing sick leave). Second, for each entity, we identified RCTs (or protocol of an RCT) with a vocational or non-vocational intervention and at least one work participation outcome. Third, for each RCT two researchers (MR and JV) determined whether the intervention was vocational or non-vocational and whether the framework could be applied to categorize the vocational outcomes. Finally, they determined whether measurement of the outcomes would meet our mandatory and optional COS criteria.

Regarding terminology, we refer to *an outcome* as the endpoint of a trial that is expected to change, such as symptoms, sick leave or return to work. We define *an outcome measurement method* as the way in which the outcome is measured e.g., through interviews, self-report, data collection from registries or questionnaires. *Outcome measurement instruments* are instruments which are used to measure the specific outcome, such as validated or self-constructed questions or questionnaires.

## Results

The search in MEDLINE (PubMed) yielded 2106 references, of which 296 were considered relevant. Most of the 1810 irrelevant references were about the application of the ICF for a specific disease and not about work participation. An additional 60 references were found through the Google search or from our personal files. Having screened these 356 references, we selected 67 for full-text analysis and included 59 articles. Fifteen studies provided a theoretical perspective of work participation based on the ICF (section A). Thirty-six studies described how to measure effectiveness of work participation interventions (section B). Eight studies provided a classification of work participation outcomes (section C). Based on the literature and the scope of our COS, we identified six mandatory criteria and two optional criteria that the framework should meet (section D). The framework we constructed includes four distinct work participation stages with relevant outcomes for each stage (section E). As a proof of concept we established a fit between the work participation outcomes of the six RCTs and the criteria of the framework (section F).

### A. Views on the concept of participation: “The International Classification of Functioning”

#### Strengths and limitations

The ICF, approved by the WHO in 2001 [26], constitutes the predominant theoretical classification of societal participation among individuals with health problems. The model is valued and broadly adopted due to its universality, comprehensiveness [27], and the capability to consider disability through a biopsychosocial perspective [28]. The ICF assesses activities and participation either on the level of having the *general capacity* to do an activity in a standardized environment or as *actual performance* within the context of their daily life [29]**.** However, the scientific community finds that the ICF is too ambiguous and incomprehensive for informing how to measure participation — its definitions do not ensure that activities and participation are mutually exclusive [30, 31]. It lacks the subjective aspect of participation (for example: satisfaction) [32, 33] and existing measurement instruments based on the ICF contain only very general questions about (work) participation [34, 35]. Moreover, the ICF codes within the (sub-) chapters do not explain the dynamics between health states, functions and how changes occur over time in the context of work participation [28, 36].

The ICF does not address the normative character of participation. Participation can be seen as performing or discharging a social role [37–40], but there is no universal standard for a “normal” level of participation. The worker role is clearly distinct from other social roles [41]. Paid work which is the focus of this project differs from volunteering work and has different normative aspects which should be considered in terms of outcome measurement.

*ICF Core Sets* are made to narrow down the list of about 1400 categories to what is most relevant to consider in practice or research for a specific setting or health problem [42]. However, such sets are not made exclusively for core outcome measurements in research and would still contain too many items. *A Core Outcome Set* must represent a minimum set of outcomes which are feasible and relevant to measure across all trials within a specific health field [16].

### B. Views on measuring work participation

#### General work participation and disability evaluation

Work participation is influenced by personal and environmental factors, such as motivation, the work environment, and national policies. The most important outcome domains or stages of work participation to be measured are context-specific. Table 1 summarizes theories of how to capture (effectiveness) results of work participation interventions. Although the ICF has been preferred as reference point, no final recommendations were made on specific critical outcomes or measurement instruments that could be used for a generic COS for Work.

**Table 1:** Perspectives of theories and models on measuring general work participation

Study	Subject	Summary
**What issues should be considered when measuring work participation**		
Iwanaga et al. [43] 2019 Goldman [44] 2013, Marfeo et al. [45] 2013, Anner et al. [28] 2012, Berglind & Gerner [46] 2002	Describe measuring (partial) disability.	Recommendations on disability evaluation which incorporated the ICF in the evaluation. Motivation and self-efficacy are important to include in disability evaluation as they are predictors of work participation.
Kim & Rhee [47] 2018	Describe how policy changes impact transitions between employment states.	Policy against disability discrimination may positively impact job retention of the (partially) disabled workers and negatively impact the inflow to the employment market of (partially) disabled and unemployed individuals.
Jetha et al. [48] 2016	Capture the complexity of work disability research.	System dynamics modelling should be applied to work participation research. Dynamic behaviours between individual, psychosocial, organizational and regulatory components need to be seen in terms of feedback loops rather than a linear process. However, this would be time consuming and complex.
Combs & Heaton [49], 2016 Sandqvist & Henriksson [50] 2004	Provide conceptual analyses of work functioning and what is important to measure.	Work participation should be seen through a holistic lens with most important components closely related to the structure of ICF.
Mehnert et al. [51] 2013	Identify which work outcomes are most important to measure for cancer survivors.	Based on existing frameworks, the following outcomes are important for cancer survivors: employment, return to work, work ability, work performance, job opportunities, income, work satisfaction, job promotion and training and sustainability in work retention.
**Use of existing models and tools to measure work participation**		
Momsen et al. [52] 2019	Operationalize ICF for vocational rehabilitation.	The ICF can be used to operationalize vocational rehabilitation. More research required to standardize the use of ICF.
Sternberg & Bethge [53] 2018, Mateen et al. [7] 2017	Review existing work outcome measures.	Evaluation of a broad range of instruments measuring work participation based on their psychometric properties. No final recommendations could be made.

#### Prevalent work participation outcomes

We reviewed the literature on specific types of work participation outcomes and constructs, as well as the methodological issues to be considered. Here, we list the most prevalent types of outcomes discussed in the order of most to least frequently measured – as found in our systematic review [10]. *Sickness absenteeism* can be defined as the decision to not attend or inability to attend work due to an illness. The decision process is phased over time and may be influenced by the supervisor - subordinate relationship, individual capacities and incentives [54–56]. *Return to work* (RtW) is influenced by factors which may differ for the stakeholders involved. In terms of RtW measurements, aspects like sensitivity to change or validity need to be considered [22, 57–63]. *Productivity* is often measured by a combination of outcomes, such as employment status, absenteeism and presenteeism. While presenteeism is receiving increasing attention in the evaluation of productivity, there is no consensus on how best to measure it. Generic measures of work performance may include measures such as task performance, counterproductive work behaviours, and adaptive performance [64–71]. *Work ability* defined as self-perceived potential for work participation, is measured mainly on the level on capacity rather than performance [72–75].

### C. Classification of work participation outcomes

Of the eight studies that provided a classification of recommended outcomes and measures for work participation, one considered the most commonly used outcome measures (including employment status, sick leave, return to work, and role functioning) [20], five used ICF items [14, 33, 42, 76, 77], and two looked at productivity [78] and absenteeism [19] separately (Table 2). A meaningful general classification of work capacity outcomes may require additional considerations, such as the aim of the study, which often revolves around the effectiveness of an intervention, or the perspective taken in the study, i.e., that of employers, workers, or society. With regard to using the ICF to operationalize work participation outcomes, some suggested using *work functioning* as an overarching term for work activities, such as driving, and *work participation* for maintaining desired employment.

**Table 2:** Current classifications for work participation outcomes

Outcome category	Study	Aim	Findings	Perspective
General work participation	Luna et al. [77] 2020	To identify measurement instruments for the ICF core set for vocational rehabilitation.	13 instruments covered 58 categories (64.5%) of the core set: 13 (76.5%) of the body functions component, 29 (72.5%) of the activities and participation component and 16 (49%) environmental factors.	Worker
	Alheresh et al. [14] 2015	To organize and define ICF-based work participation outcomes.	Definitions for disability, activity, participation, activity limitations, and participation restrictions.	Worker, employer, societal, economic
	Finger et al. [42] 2012	Brief ICF core set important for vocational rehabilitation.	Consensus about brief Core Set including 13 ICF categorized items: 6 for activities and participation, 4 for environmental factors, 3 for body functions.	Healthcare
	Escorpizo et al. [76] 2011	List of ICF items relevant for vocational rehabilitation.	The following 101 ICF categories were listed as relevant for work participation: 22 for body functions, 13 for body structures, 36 for activities and participation, and 30 for environmental factors.	Healthcare
	Glässel et al. [33] 2011	List of ICF items relevant for patients in vocational rehabilitation.	List contains 160 ICF categories. ICF components (a) body functions, (b) activities and participation and (c) environmental factors were equally represented, while (d) body structures appeared less frequently.	Worker
	Amick et al. [20] 2000	To review and illustrate a sample of work outcome measures.	Five reasons and measures for work outcomes: 1. Productivity loss in clinical trials; 2. Effects of health services; 3. Effects of injury prevention; 4. Effects of work reorganization, such as ergonomic changes; 5. Improvement of the provider – worker interaction.	Worker, employer, societal, economic
Productivity	Beaton et al. [78] 2016	To recommend OMERACT productivity measures for Rheumatoid Arthritis.	Provisional recommendations: WALS (Workplace Activity Limitations Scale), WLQ-25 PDmod (Work Limitations Questionnaire with modified physical demands scale), WAI (Work Ability Index), WPS (Arthritis-specific Work Productivity Survey), and WPAI (Work Productivity and Activity Impairment Questionnaire).	Patient, economic
Absenteeism	Hensing et al. [19] 1998	To examine sick leave measures used in research.	Five measures for sick leave spells are recommended: frequency, length, incidence rate, cumulative incidence and duration.	Epidemiological

*Work disability* may mean a limitation on work activities, such as difficulty in driving, or a participation restriction, such as the number of hours lost from work. The relationship between participation and disability can be influenced by contextual factors, and people can move in and out of limitations and restrictions over time. *Restrictions* on w*ork participation* include restrictions in fulfilling the worker-role, expressed as a lesser than desired status in work productivity, employment status, career advancement or job opportunities [14].

Researchers have proposed varying lists of ICF items [33, 42, 76] and measurement instruments that could be used to measure a core set of ICF items for vocational rehabilitation [77]. However, these lists address a broad spectrum of issues related to vocational rehabilitation beyond outcomes that measure the effectiveness of interventions and do not intend to be a classification of outcomes to be used in trials.

A recent COS for rheumatoid arthritis evaluated measurement instruments for at-work productivity loss which included absenteeism and presenteeism, measured as ‘number of days or hours off work’, or ‘difficulties at work’ [78]. However, currently there is no agreed measure of presenteeism which is underpinned by economic theory [68, 69, 79]. For measuring sick leave, five measures of sick leave spells have been suggested: their frequency, length, incidence rate, cumulative incidence, and duration [19, 80, 81].

### D. Criteria used by the framework for work participation outcomes

Our discussions concluded that not all criteria are equally relevant and feasible to measure for interventions with varying aims. Therefore, we distinguished between six mandatory and two optional criteria (Table 3). In addition, outcome measures should capture transitions in work participation [55, 63] in which people with a health problem seek work, are absent from work, or who are at risk of losing their jobs. This criterion applies to the COS as a whole, because no single outcome captures transitions between work phases.

**Table 3:** COS Criteria: outcomes used in COS for Work should meet these criteria

Outcomes should	Reason for inclusion
*Mandatory criteria*	
1. Be sensitive to change	The aim of using a COS is to compare outcomes of intervention studies [16]. This implies that the measures of the outcomes need to be sensitive to change [61, 80] for any type of intervention study which may impact work participation.
2. Be feasible to measure	COS outcomes should represent a minimal set of outcomes that can be measured [16].
3. Be applicable internationally	COS are developed for international use to make large scale evidence synthesis possible [16].
4. Be specific for work participation	Outcomes should relate to paid work to address specific factors of the worker role that are not transferable to voluntary work [41].
5. Capture the perspectives of multiple stakeholders	Work participation outcomes are of relevance for people who (aim to) work, employers, policy maker, health professionals, and researchers [47–50].
6. Be in alignment with the ICF model	The ICF is a widely used model in (occupational) health sciences and practice [13, 14, 27, 28, 33, 77].
*Optional criteria*	
7. Be used for cost-effectiveness studies	Cost-effectiveness analysis is important for societal decision making.
8. Be applicable across different insurance schemes	As COS for Work is intended to be applicable internationally, the outcomes should be as relevant as possible, irrespective of different insurance schemes.

### E. Framework for work participation outcomes

Using the ICF, we established four stages of work participation that should help identify outcomes that potentially fit into a COS for Work: *Stage 1: Initiating employment; Stage 2: Having employment; Stage 3: Increasing or maintaining productivity at work; Stage 4: Return to employment.* These stages do not represent an order but represent different situations that inform whether a particular stage is applicable and can be used to select an outcome.

#### Stage 1: initiating employment

Outcomes relevant for work participation within this stage help determine whether participants are ready for initiating employment. ICF categories “apprenticeship” (work preparation)” and “acquiring a job” apply. The target group is unemployed at baseline (not self-employed or contracted by an employer, but possibly with a type of subsidized governmental wage replacement benefit). The intervention types are commonly vocational, such as Individual Placement and Support programs which help people with a chronic mental health problem to gain work [10]. They focus on increasing skills, knowledge, or attitude of participants for successful engagement in a worker role. Outcomes could measure time to first job, readiness for work, motivation for work, or job seeking skills (Table 4).

**Table 4:** Work participation stage “Initiating employment”

Target group	Type of intervention	Examples of outcomes
- Unemployed individuals aiming to get work	- Vocational interventions helping people with a health problem to gain employment	- Time to first job - Readiness for work - Motivation for work - Job seeking skills - Job interview skills

#### Stage 2: having employment

Outcomes relevant for this stage of work participation indicate whether a person is in employment, can retain employment or loses employment within the duration of the intervention study. These outcomes fit the ICF categories “remunerative employment” or “keeping a job”. Having employment entails producing goods or services in exchange for a monetary compensation, salary or wage – with an employer or as self-employed [82, 83]. Unemployment can be seen as involuntary joblessness [84]. The target group are people at risk of losing employment due to a health problem, like cancer survivors [51, 85], patients with cardiovascular disease or diabetes [86] whose disease status is associated with a higher unemployment rate. At the start of interventions persons for whom it is difficult to become employed may also progress from unemployment to having employment – in such cases it is also important to have measures on whether and for how long the participants were employed.

Relevant interventions can be vocational or non-vocational (Table 5). Vocational rehabilitation studies commonly have a primary aim to investigate the effect of an intervention on employment status, while medical or pharmaceutical studies tend to measure the effect of the intervention (often a new drug) on employment status as a secondary outcome. The outcomes within this stage pertain to the employment status of the individual for the duration of the study.

**Table 5:** Work participation stage “Having employment”

Target group	Type of intervention	Examples of outcomes
- Unemployed individuals aiming to get work - Employed persons at risk of losing employment	- Vocational interventions aiming to help people gain work or prevent people from losing work - Non-vocational interventions	- Employment rate (part/full time) - Employment duration - Job loss - Early retirement due to ill health

#### Stage 3: increasing or maintaining productivity at work

Outcomes relevant to this stage of work participation refer to people who experience limitations / restrictions with working or have less output, i.e. loss of productivity. In terms of ICF, the outcomes could be placed under the category “maintaining a job”. The target group are individuals holding a job and experiencing functional problems at work due to health problems. Interventions could address vocational measures such as providing work related rehabilitation, or non-vocational interventions such as medical drugs. Both types of interventions may impact outcomes such as: work ability, work functioning, work impairment or overall productivity (loss) (Table 6). The latter is often used to calculate costs [67, 69]. From the worker perspective, feeling fit to work is essential for a successful work life. To this end, employers can provide preventive measures, aiming to reduce stress and increase wellbeing at work [87, 88].

**Table 6:** Work participation stage “Increasing or maintaining productivity at work”

Target group	Type of intervention	Examples of outcomes
- Individuals holding a job and experiencing functional problems at work due to a health problem	- Vocational interventions providing work related rehabilitation - Work related vitality interventions - Non-vocational interventions which may also impact at-work functioning	- At-work productivity loss - Work ability - Work activity impairment - Vitality at work

#### Stage 4: return to employment

Outcomes relevant for this stage of work participation determine whether people who have been (temporarily) unable to work and have been on sick leave, successfully resume work. We thereby consider absenteeism (e.g., sick leave) and return to work as measures from different perspectives, but belonging to the same concept of not working (fully) in spite of having employment. The ICF does not provide a category sickness absence, but outcomes for this stage could fit under the ICF categories “maintaining employment” and “remunerative employment”.

Sickness absence can show that a person is unable to fulfil their worker role due to ill health, but other reasons for absenteeism, such as maternity leave or unwillingness to come to work also exist. Vocational rehabilitation designed to help workers return to work includes outcomes of work participation as primary outcome. But any type of clinical, pharmaceutical or otherwise health related intervention may indirectly impact outcomes relevant for this stage (Table 7). Common outcomes are return to work rate, time to return to work, sick leave rate, sick leave duration or frequency. Others report perceived capacity to return to work, such as intention to return to work, return to work self-efficacy or the need for recovery from work.

**Table 7:** Work participation stage “Return to employment”

Target group	Type of intervention	Examples of outcomes
- Individuals holding a job but not working (fully) due to health reasons	- Non-vocational interventions which may impact sick leave (clinical, pharmacological) - Vocational interventions aiming to help people get back to work	- Return to work rate (part/fulltime) - Time to return to work - Sick leave rate - Sick leave duration - Intention to return to work - Return to work self-efficacy - Need for recovery

More recently, ‘return to work’ has been introduced as an indicator of sickness absence with an individual perspective, when the worker role is important for economic reasons and for reasons of well-being, e.g., when cancer survivors report that getting back to work is a final step in getting back to normal life after their disease and treatment experience [51].

Absenteeism outcomes are frequently approached from a societal or economic perspective [89]. Importantly, sick leave time can be converted into monetary value as part of economic evaluations to indicate extra costs for the employer or/and the employee.

##### Disease trajectories and stages of work participation

**Fig. 2:**
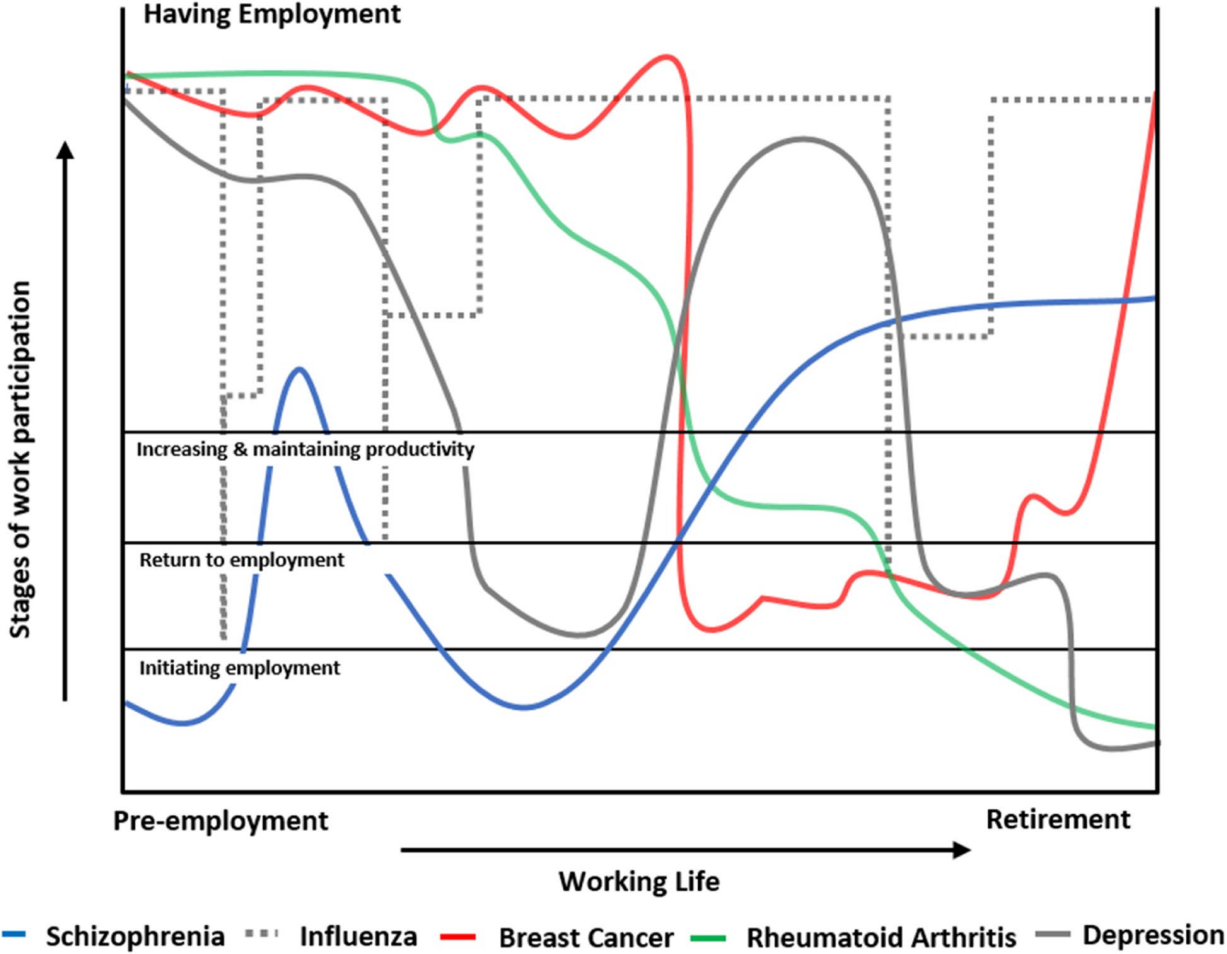
Possible work participation trajectories with the y-axis indicating the severity of disruption of work participation and the x-axis indicating time from start to end of working life. The health problem that affects the worker is an important indicator of how people move through the four stages of work participation during their working life. Influenza hardly influences work participation but schizophrenia severely disrupts work participation

Different disease trajectories may predetermine which stages of work participation the outcome measurement should focus on, as illustrated in Fig. 2 which includes five common diseases [90–95]. Besides the disease status or its course of progression (chronic, progressive, intermittent, relapsing, resolving), the baseline status of the target group (employed-unemployed, seeking-maintaining-losing work) may determine the preferred outcome, and the specific intervention types or aims [11].

### F. Feasibility of the framework as a proof of concept

The predefined COS criteria could be applied to almost all measured outcomes in the six RCTs (Table 8) that we selected to evaluate proof of concept. Since we had to take a number of assumptions, some caveats apply:

**Table 8:** Proof of concept for the framework. We applied the COS criteria for outcomes on work participation (Table 3) to the work outcomes used in 6 RCTs with different health conditions and heterogeneous disability trajectories. We compared the extent to which work participation outcomes in RCTs met the 8 COS criteria

Participants; Health condition	Participants; employment status at inclusion	Work participation stage	Intervention	Measured work participation outcomes	Fit with mandatory criteria						Fit with optional criteria	
					1	2	3	4	5	6	7	8
Schizophrenia [96]	Unemployed	Initiating employment, Having employment	Vocational rehabilitation	-Frequency of job interviews, -Work status, -Worked hours	x	x	x	x	x	x		x
Rheumatoid arthritis [97]	40% employed	Increasing or maintaining productivity at work	Pharmaceutical	-Work productivity	x	x	x	x	x	x		x
Depression [98]	83% employed	Return to employment	Using self-assessment of depression course during GP consultations	-Work ability, −Sick leave, -Time to return to work	x	x	x	x	x	x	x	x
Breast cancer [99]	Unspecified	Return to employment	Post radiation exercise program	-Return to work (sick leave)	x	x	x	x	x	x	x	x
Influenza [100]	100% employed	Return to employment	Vaccination against influenza	-Sick leave	x	x	x	x	x	x	x	x
On sick leave for at least 6 months due to any health problem [101]	Employed	Return to employment	Independent medical evaluation	-Sick leave	x	x	x	x	x	x	x	x

*Legend*: COS criteria: Mandatory criteria 1: be sensitive to change; 2: be feasible to measure; 3: be applicable internationally; 4: be work participation specific; 5: capture multiple stakeholder perspectives; 6: be in alignment with the ICF model. Optional criteria 7: be used for cost-effectiveness studies; 8: be applicable across different insurance schemes

For criteria 1 (sensitive to change) and 2 (feasible to measure), we assumed that the outcomes measured in the studies fulfilled these criteria, without confirmation from robust evidence. We considered criterion 5 (capture multiple stakeholder perspective) to be met, if the outcomes were considered relevant for at least two stakeholders, based on face validity. It was not easy to match the outcomes with the ICF (criterion 6). There are no specific ICF codes for absenteeism, work ability, and productivity, but we have assigned these terms to related ICF codes, as detailed above.

Optional criteria: It is not clear which outcome measures can be reliably used in economic analyses [68, 69, 79]. Because sick leave data are the most commonly used [10] we assumed that this outcome meets the optional criterion 7 (use of outcome for cost-effectiveness analysis). Finally, our assumption that all outcomes can be transferred to different insurance schemes (criterion 8) seems plausible, but this needs further evaluation.

## Discussion

Work participation can best be considered as the engagement in a major life area that is important for most persons and as discharging the worker role. The ICF is a leading framework in defining work functioning, activities and participation, and its counterpart work disability. Terminology of outcomes require critical attention because standardization is lacking. There is no classification of outcomes available that fulfills multiple criteria for a COS on work participation. In our framework we propose four comprehensive work participation stages that help to select work outcomes: (1) initiating employment, (2) having employment, (3) increasing or maintaining productivity at work and (4) return to employment.

The studies included in this paper contain theories and findings on what is important to consider for the assessment of work-participation in general [5, 14, 20, 26, 31, 34, 46, 48, 51–53, 76, 102], and in some cases specifically for an outcome category, such as productivity or sick leave [65, 67]. Theories describing the general work-participation process mention a broad range of factors which could be relevant to include in studies which measure improvements in work participation. However, no clear prioritization is indicated on the level of outcomes and consideration of the temporal aspect is often lacking.

We evaluated theories and studies based on empirical findings on what is currently considered as relevant for the assessment of work participation. Our proposed framework is novel in terms of structuring the work participation process on the level of most critical outcomes, including the temporal aspect of various disease courses and progression within the different stages of work-participation. Various stakeholder perspectives are important to consider when measuring work participation. However, prioritization of outcomes may vary per stakeholder group [57]. This should be further investigated.

Using the proof of concept approach we applied various disease courses and work participation stages reported in trials with varying aims and showed that work participation outcomes fit within our framework. However, the framework suggests a broader scope of outcomes than what trialists may be used to consider. For example, studies on return to work typically do not include outcomes such as “RtW self-efficacy”. Looking at literature, such an outcome could be considered for a COS as it could be an important indicator of sustainable RtW. Subjective outcomes are most likely to be under researched and only included in vocational trials [10].

Although we used a sensitive search strategy for our systematic literature review, it is possible that we did not include all theories or frameworks which are also used in research and practice. In addition, our proof of concept is applied to six types of studies which could mean that not all possible outcomes may have been considered. Last, it is difficult to ascertain whether the four stages of work participation are applicable on an international scale because of possible variation of various regulatory approaches across countries. Statutory regulations influence what is prioritized in occupational health in an continuously evolving manner [103] and indirectly dictate definitions and terminology of (work participation) concepts- such as what it means to be (un)employed [104]. A more elaborate analysis of international policy and work outcome measurements is needed to show which outcomes categories are equally applicable across countries with different regulatory regulations.

We will build on the proposed stages of work participation, each with distinct and corresponding types of outcomes, for the further development of a COS for Work. This will involve reaching consensus amongst stakeholders on what type of outcomes are essential to be measured for each stage. Consensus will specifically need to focus on unambiguous outcome definitions for measuring: employment status in stage 2, decreased performance on the job in stage 3, sickness absence and RtW in category 4 and if subjective outcomes could be relevant for broad use such as *motivation for work* and *RtW self-efficacy*. Further, clinimetrically sound measurement methods need to be determined for the set of outcomes, ensuring feasibility for international and cross-disciplinary research.

The COS for Work will enable researchers to compare data on a larger scale and draw better conclusions on which interventions are most effective in promoting work participation. Use of COS will also help reduce research waste and assist policy makers and practitioners in making better informed decisions on worthwhile investments.

## Concluding remarks

We present a framework for selecting work-participation outcome measurements which will be used for the development of a COS. The framework is presented from the perspective of various stages of the work participation process, and it can be applied to any type of participant population, aim of intervention and by the international community. We propose the following four stages of work participation: (1) initiating employment, (2) having employment, (3) increasing or maintaining productivity at work and (4) return to employment.

## Supplementary Information

**Additional file 1: search strategy**.

## Data Availability

All data generated or analyzed during this study are included in this published article.

## References

[1] World Health Organization (2011). World report on disability.

[2] Tough H, Siegrist J, Fekete C (2017). Social relationships, mental health and wellbeing in physical disability: a systematic review. BMC Public Health.

[3] Varekamp I, Verbeek JH, van Dijk FJ (2006). How can we help employees with chronic diseases to stay at work? A review of interventions aimed at job retention and based on an empowerment perspective. Int Arch Occup Environ Health.

[4] Higgins JP, Thomas J, Chandler J, Cumpston M, Li T, Page MJ (2019). Cochrane handbook for systematic reviews of interventions.

[5] Arends I, Bruinvels DJ, Rebergen DS, Nieuwenhuijsen K, Madan I, Neumeyer-Gromen A, et al. Interventions to facilitate return to work in adults with adjustment disorders. Cochrane Database Syst Rev. 2012;12:Art. no. CD006389. 10.1002/14651858.CD006389.pub2 PMC11627150 23235630

[6] Hoving JL, Lacaille D, Urquhart DM, Hannu TJ, Sluiter JK, Frings-Dresen MH. Non-pharmacological interventions for preventing job loss in workers with inflammatory arthritis. Cochrane Database Syst Rev. 2014;11:Art. no CD010208. 10.1002/14651858.CD010208.pub2 PMC11287239 25375291

[7] Mateen BA, Doogan C, Hayward K, Hourihan S, Hurford J, Playford ED (2017). Systematic review of health-related work outcome measures and quality criteria-based evaluations of their psychometric properties. Arch Phys Med Rehabil.

[8] Schaafsma FG, Whelan K, van der Beek AJ, van der Es-Lambeek LC, Ojajärvi A, Verbeek JH. Physical conditioning as part of a return to work strategy to reduce sickness absence for workers with back pain. Cochrane Database Syst Rev. 2013;8:Art. no. CD001822. 10.1002/14651858.CD001822.pub3 PMC7074637 23990391

[9] van Oostrom SH, Driessen MT, de Vet HC, Franche RL, Schonstein E, Loisel P, et al. Workplace interventions for preventing work disability. Cochrane Database Syst Rev. 2009;2:Art. no. CD006955. 10.1002/14651858.CD006955.pub2 19370664

[10] Ravinskaya M, Verbeek JH, Langendam M, Daams JG, Hulshof CT, Madan I (2022). Extensive variability of work participation outcomes measured in randomized controlled trials: a systematic review. J Clin Epidemiol.

[11] Ravinskaya M, Verbeek JH, Langendam MW, Madan I, Verstappen SM, Kunz R, et al. Preferred methods of measuring work participation: an international survey among Trialists and Cochrane systematic reviewers. J Occup Rehabil. 2022;1–9:620–28. 10.1007/s10926-022-10031-0 PMC9668767 35347539

[12] Rosenbaum P, Stewart D. The World Health Organization International Classification of Functioning, Disability, and Health: a model to guide clinical thinking, practice and research in the field of cerebral palsy. Semin Pediatr Neurol. 2004; Elsevier; 11(1):5–10. 10.1016/j.spen.2004.01.002 15132248

[13] Escorpizo R, Reneman MF, Ekholm J, Fritz J, Krupa T, Marnetoft SU (2011). A conceptual definition of vocational rehabilitation based on the ICF: building a shared global model. J Occup Rehabil.

[14] AlHeresh RA, Keysor JJ (2015). The work activity and participation outcomes framework: a new look at work disability outcomes through the lens of the ICF. Int J Rehabil Res.

[15] Kirkham JJ, Gargon E, Clarke M, Williamson PR (2013). Can a core outcome set improve the quality of systematic reviews?–a survey of the co-ordinating editors of Cochrane review groups. Trials.

[16] Williamson PR, Altman DG, Bagley H, Barnes KL, Blazeby JM, Brookes ST (2017). The COMET handbook: version 1.0. Trials.

[17] Tang K, Boonen A, Verstappen SM, Escorpizo R, Luime JJ, Lacaille D (2014). Worker productivity outcome measures: OMERACT filter evidence and agenda for future research. J Rheumatol.

[18] Kirkham JJ, Clarke M, Williamson PR (2017). A methodological approach for assessing the uptake of core outcome sets using ClinicalTrials. Gov: findings from a review of randomised controlled trials of rheumatoid arthritis. BMJ.

[19] Hensing G, Alexanderson K, Allebeck P, Bjurulf P (1998). How to measure sickness absence? Literature review and suggestion of five basic measures. Scand J Soc Med.

[20] Amick BC, Lerner D, Rogers WH, Rooney T, Katz JN (2000). A review of health-related work outcome measures and their uses, and recommended measures. Spine (Phila Pa 1976).

[21] Lerner D, Amick BC III, Rogers WH, Malspeis S, Bungay K, Cynn D. The work limitations questionnaire. Med Care. 2001;39(1):72–85. 10.1097/00005650-200101000-00009 11176545

[22] Young AE, Wasiak R, Roessler RT, McPherson KM, Anema JR, van Poppel MN (2005). Return-to-work outcomes following work disability: stakeholder motivations, interests and concerns. J Occup Rehabil.

[23] Beaton D, Bombardier C, Escorpizo R, Zhang W, Lacaille D, Boonen A (2009). Measuring worker productivity: frameworks and measures. J Rheumatol.

[24] Kendig CE (2016). What is proof of concept research and how does it generate epistemic and ethical categories for future scientific practice?. Sci Eng Ethics.

[25] Jaakkola E (2020). Designing conceptual articles: four approaches. AMS Rev.

[26] World Health Organization (2001). International classification of functioning, disability and health.

[27] Martins AC (2015). Using the international classification of functioning, disability and health (ICF) to address facilitators and barriers to participation at work. Work.

[28] Anner J, Schwegler U, Kunz R, Trezzini B, de Boer W (2012). Evaluation of work disability and the international classification of functioning, disability and health: what to expect and what not. BMC Public Health.

[29] Stephens D (2001). World Health International Classification of Functioning, Disability and Health--ICF. J Audiol Med.

[30] Dijkers MP (2010). Issues in the conceptualization and measurement of participation: an overview. Arch Phys Med Rehabil.

[31] Nowak LL, Davis AM, Mamdani M, Beaton D, Schemitsch EH. A concept analysis and overview of outcome measures used for evaluating patients with proximal humerus fractures. Disabil Rehabil. 2019;43(10):1–13. 10.1080/09638288.2019.1649728 31479302

[32] Hemmingsson H, Jonsson H (2005). An occupational perspective on the concept of participation in the International classification of functioning, disability and health--some critical remarks. Am J Occup Ther.

[33] Glässel A, Finger ME, Cieza A, Treitler C, Coenen M, Escorpizo R (2011). Vocational rehabilitation from the client's perspective using the international classification of functioning, disability and health (ICF) as a reference. J Occup Rehabil.

[34] Resnik L, Plow MA (2009). Measuring participation as defined by the international classification of functioning, disability and health: an evaluation of existing measures. Arch Phys Med Rehabil.

[35] Ballert CS, Hopfe M, Kus S, Mader L, Prodinger B (2019). Using the refined ICF linking rules to compare the content of existing instruments and assessments: a systematic review and exemplary analysis of instruments measuring participation. Disabil Rehabil.

[36] Pransky G (2013). Measurement of outcomes in WDP: conceptual and methodological considerations and recommendations for measuring outcomes. Handbook of work disability.

[37] Sciaraffa S (2011). Identification, meaning, and the normativity of social roles. Eur J Philos.

[38] James WB, Witte JE, Galbraith MW (2006). Havighurst’s social roles revisited. J Adult Dev.

[39] Mechanic D (1962). The concept of illness behavior. J Chronic Dis.

[40] Mechanic D (1995). Sociological dimensions of illness behavior. Soc Sci Med.

[41] Chang FH, Coster WJ (2014). Conceptualizing the construct of participation in adults with disabilities. Arch Phys Med Rehabil.

[42] Finger ME, Escorpizo R, Glässel A, Gmünder HP, Lückenkemper M, Chan C (2012). ICF Core set for vocational rehabilitation: results of an international consensus conference. Disabil Rehabil.

[43] Iwanaga K, Chan F, Tansey TN, Strauser D, Ritter E, Bishop M (2019). Working Alliance and stages of change for employment: the intermediary role of autonomous motivation, outcome expectancy and vocational rehabilitation engagement. J Occup Rehabil.

[44] Goldman HH (2013). Commentary on measuring disability. Arch Phys Med Rehabil.

[45] Marfeo EE, Haley SM, Jette AM, Eisen SV, Ni P, Bogusz K (2013). Conceptual foundation for measures of physical function and behavioral health function for social security work disability evaluation. Arch Phys Med Rehabil.

[46] Berglind H, Gerner U (2002). Motivation and return to work among the long-term sicklisted: an action theory perspective. Disabil Rehabil.

[47] Kim S, Rhee S (2018). Measuring the effects of employment protection policies: theory and evidence from the Americans with disabilities act. Labour Econ.

[48] Jetha A, Pransky G, Hettinger LJ (2016). Capturing complexity in work disability research: application of system dynamics modeling methodology. Disabil Rehabil.

[49] Combs B, Heaton K (2016). Occupational functionality: a concept analysis. Workplace Health Saf.

[50] Sandqvist JL, Henriksson CM (2004). Work functioning: a conceptual framework. Work.

[51] Mehnert A, de Boer A, Feuerstein M (2013). Employment challenges for cancer survivors. Cancer.

[52] Momsen AH, Stapelfeldt CM, Rosbjerg R, Escorpizo R, Labriola M, Bjerrum M (2019). International classification of functioning, disability and health in vocational rehabilitation: a scoping review of the state of the field. J Occup Rehabil.

[53] Sternberg A, Bethge M (2018). Measuring work functioning in individuals with musculoskeletal disorders with reference to the international classification of functioning, disability, and health: a systematic literature review. Int J Rehabil Res.

[54] Halbesleben JRB, Whitman MV, Crawford WS (2014). A dialectical theory of the decision to go to work: bringing together absenteeism and presenteeism. Hum Resour Manag Rev.

[55] Labriola M (2008). Conceptual framework of sickness absence and return to work, focusing on both the individual and the contextual level. Work.

[56] Thulesius HO, Grahn BE (2007). Reincentivizing-a new theory of work and work absence. BMC Health Serv Res.

[57] Hees HL, Nieuwenhuijsen K, Koeter MW, Bultmann U, Schene AH (2012). Towards a new definition of return-to-work outcomes in common mental disorders from a multi-stakeholder perspective. PLoS One.

[58] Pransky G, Gatchel R, Linton SJ, Loisel P (2005). Improving return to work research. J Occup Rehabil.

[59] Leyshon R, Shaw L (2012). Using multiple stakeholders to define a successful return to work: a concept mapping approach. Work.

[60] Shaw WS, Linton SJ, Pransky G (2006). Reducing sickness absence from work due to low back pain: how well do intervention strategies match modifiable risk factors?. J Occup Rehabil.

[61] Butler JR, Johnson WG, Baldwin ML. Managing work disability: why first return to works is not a measure of success. ILR Review. 1995;48(3):452–69.

[62] Claudi Jensen AG (2013). Towards a parsimonious program theory of return to work intervention. Work.

[63] Krause N, Frank JW, Dasinger LK, Sullivan TJ, Sinclair SJ (2001). Determinants of duration of disability and return-to-work after work-related injury and illness: challenges for future research. Am J Ind Med.

[64] Verstappen SM, Fautrel B, Dadoun S, Symmons DP, Boonen A (2012). Methodological issues when measuring paid productivity loss in patients with arthritis using biologic therapies: an overview of the literature. Rheumatology (Oxford).

[65] Navarro A, Salas-Nicas S, Llorens C, Moncada S, Molinero-Ruiz E, Morina D (2019). Sickness presenteeism: are we sure about what we are studying? A research based on a literature review and an empirical illustration. Am J Ind Med.

[66] Rainbow JG, Steege LM (2017). Presenteeism in nursing: an evolutionary concept analysis. Nurs Outlook.

[67] Koopmanschap M, Burdorf A, Jacob K, Meerding WJ, Brouwer W, Severens H (2005). Measuring productivity changes in economic evaluation: setting the research agenda. Pharmacoeconomics.

[68] Jones C, Payne K, Gannon B, Verstappen S (2016). Economic theory and self-reported measures of Presenteeism in musculoskeletal disease. Curr Rheumatol Rep.

[69] Jones C, Verstappen SMM, Payne K (2019). A systematic review of productivity in economic evaluations of workplace interventions: a need for reporting criteria?. Appl Health Econ Health Policy.

[70] Mattke S, Balakrishnan A, Bergamo G, Newberry SJ (2007). A review of methods to measure health-related productivity loss. Am J Manag Care.

[71] Koopmans L, Bernaards CM, Hildebrandt VH, Schaufeli WB, de Vet Henrica CW, van der Beek AJ (2011). Conceptual frameworks of individual work performance: a systematic review. J Occup Environ Med.

[72] Ilmarinen J (2009). Work ability-a comprehensive concept for occupational health research and prevention. Scand J Work Environ Health.

[73] Metzinger C, Berg C (2015). Work readiness tools for young adults with chronic conditions. Work.

[74] Tengland PA (2011). The concept of work ability. J Occup Rehabil.

[75] Jansson I, Björklund A, Perseius KI, Gunnarsson AB (2015). The concept of ‘work ability’ from the view point of employers. Work.

[76] Escorpizo R, Finger ME, Glässel A, Gradinger F, Lückenkemper M, Cieza A (2011). A systematic review of functioning in vocational rehabilitation using the international classification of functioning. Disabil Health J Occup Rehabil.

[77] Luna JS, Monteiro GTR, Koifman RJ, Bergmann A (2020). International classification of functioning in professional rehabilitation: instruments for assessing work disability. Rev Saude Publica.

[78] Beaton DE, Dyer S, Boonen A, Verstappen SM, Escorpizo R, Lacaille DV (2016). OMERACT filter evidence supporting the measurement of at-work productivity loss as an outcome measure in rheumatology research. J Rheumatol.

[79] Gardner BT, Dale AM, Buckner-Petty S, Van Dillen L, Amick BC, Evanoff B (2016). Comparison of employer productivity metrics to lost productivity estimated by commonly used questionnaires. J Occup Environ Med.

[80] Hensing G (2004). Swedish council on technology assessment in health care (SBU). Chapter 4. Methodological aspects in sickness-absence research. Scand J Public Health Suppl.

[81] Hensing G (2009). The measurements of sickness absence; a theoretical perspective. Norsk Epidemiologi.

[82] Boonen A, Putrik P, Marques ML, Alunno A, Abasolo L, Beaton D (2021). EULAR points to consider (PtC) for designing, analysing and reporting of studies with work participation as an outcome domain in patients with inflammatory arthritis. Ann Rheum Dis.

[83] Boonen A, Webers C, Butink M, Barten B, Betteridge N, Black DC, et al. 2021 EULAR points to consider to support people with rheumatic and musculoskeletal diseases to participate in healthy and sustainable paid work. Ann Rheum Dis. 2022;0:1–8. 10.1136/ard-2022-222678 36109139

[84] Sawyer M, Spencer D (2008). On the definition of involuntary unemployment. J Socio-Econ.

[85] De Boer AG, Taskila T, Ojajärvi A, Van Dijk FJ, Verbeek JH (2009). Cancer survivors and unemployment: a meta-analysis and meta-regression. JAMA.

[86] Kouwenhoven-Pasmooij T, Burdorf A, Roos-Hesselink J, Hunink M, Robroek S (2016). Cardiovascular disease, diabetes and early exit from paid employment in Europe; the impact of work-related factors. Int J Cardiol.

[87] Abdin S, Welch R, Byron-Daniel J, Meyrick J (2018). The effectiveness of physical activity interventions in improving well-being across office-based workplace settings: a systematic review. Public Health.

[88] Bartlett L, Martin A, Neil AL, Memish K, Otahal P, Kilpatrick M (2019). A systematic review and meta-analysis of workplace mindfulness training randomized controlled trials. J Occup Health Psychol.

[89] Boccuzzi SJ (2003). Indirect health care costs. Cardiovascular health care economics.

[90] Hakulinen C, McGrath JJ, Timmerman A, Skipper N, Mortensen PB, Pedersen CB (2019). The association between early-onset schizophrenia with employment, income, education, and cohabitation status: nationwide study with 35 years of follow-up. Soc Psychiatry Psychiatr Epidemiol.

[91] Kalyani RR, Ji N, Carnethon M, Bertoni AG, Selvin E, Gregg EW (2017). Diabetes, depressive symptoms, and functional disability in African Americans: the Jackson heart study. J Diabetes Complicat.

[92] Tamminga SJ, Verbeek JH, de Boer AG, van der Bij RM, Frings-Dresen MH (2013). A work-directed intervention to enhance the return to work of employees with cancer: a case study. Work.

[93] Hansen S, Zimmerman P-A, van de Mortel TF (2018). Infectious illness prevention and control methods and their effectiveness in non-health workplaces: an integrated literature review. J Infect Prev.

[94] Hansen SM, Hetland ML, Pedersen J, Østergaard M, Rubak TS, Bjorner JB (2017). Work ability in rheumatoid arthritis patients: a register study on the prospective risk of exclusion and probability of returning to work. Rheumatology.

[95] Chi W-C, Yen C-F, Liou T-H, Chang K-H, Liao H-F, Chang Y-L (2021). Exploring factors associated with functional change and predictors of participation improvement—a two years follow-up on people with depression. Int J Environ Res Public Health.

[96] Zhang GF, Tsui CM, Lu AJB, Yu LB, Tsang HWH, Li D (2017). Integrated supported employment for people with schizophrenia in mainland China: a randomized controlled trial. Am J Occup Ther.

[97] Strand V, Gossec L, Proudfoot CW, Chen C-I, Reaney M, Guillonneau S (2018). Patient-reported outcomes from a randomized phase III trial of sarilumab monotherapy versus adalimumab monotherapy in patients with rheumatoid arthritis. Arthr Res Ther.

[98] Petersson E, Wikberg C, Westman J, Ariai N, Nejati S, Björkelund C (2018). Effects on work ability, job strain and quality of life of monitoring depression using a self-assessment instrument in recurrent general practitioner consultations: a randomized controlled study. Work.

[99] Ibrahim M, Muanza T, Smirnow N, Sateren W, Fournier B, Kavan P (2017). Time course of upper limb function and return-to-work post-radiotherapy in young adults with breast cancer: a pilot randomized control trial on effects of targeted exercise program. J Cancer Surviv.

[100] Nichol KL, Lind A, Margolis KL, Murdoch M, McFadden R, Hauge M (1995). The effectiveness of vaccination against influenza in healthy, working adults. N Engl J Med.

[101] Husabo E, Monstad K, Holmås TH, Oyeflaten I, Werner EL, Maeland S (2017). Protocol for the effect evaluation of independent medical evaluation after six months sick leave: a randomized controlled trial of independent medical evaluation versus treatment as usual in Norway. BMC Public Health.

[102] Dijkers MP, Hart T, Tsaousides T, Whyte J, Zanca JM (2014). Treatment taxonomy for rehabilitation: past, present, and prospects. Arch Phys Med Rehabil.

[103] Leka S, Jain A (2020). Surveillance, monitoring, and evaluation: regulatory and voluntary approaches on health, safety, and well-being. Handbook of disability, work and health.

[104] Baxandall P (2002). Explaining differences in the political meaning of unemployment across time and space. J Socio-Econ.

