# Supplementary material for: A general framework for selecting work participation outcomes in intervention studies among persons with health problems: a concept paper
Source: BMC Public Health. 2022 Nov 26;22:2189. doi: 10.1186/s12889-022-14564-0 (PMC9701431; doi:10.1186/s12889-022-14564-0)
Supplement: Supplementary file 1 — Additional file 1: search strategy. [file 12889_2022_14564_MOESM1_ESM.docx]

**Appendix 1**

To develop the search strategy, we used search terms for the concept of work participation and for framework.

# 1 “Work participation”[ti] OR workparticipation[ti] OR “sick leave”[ti] OR sickleave[ti] OR absenteeism[ti] OR presenteeism[ti] OR “work disability”[ti] OR workdisability[ti] OR “work ability”[ti] OR vocational[ti] OR functioning[ti] OR impairments[ti] OR productivity[ti] OR employment[ti] OR “return to work”[ti]

#2 “Work participation”[ot] OR “sick leave”[ot] OR absenteeism[ot] OR presenteeism[ot] OR “work disability”[ot] OR “work ability”[ot] OR vocational[ot] OR functioning[ot] OR impairments[ot] OR productivity[ot] OR employment[ot] OR “return to work”[ot]

#3 framework[ti] OR classification[ti] OR methodological[ti] OR theory[ti] OR concept[ti]

#4 framework[ot] OR classification[ot] OR methodological[ot] OR theory[ot] OR concept[ot]

#5 (#1 OR #2) AND (#3 OR #4)
